# Liver manifestations in a cohort of 39 patients with congenital disorders of glycosylation: pin-pointing the characteristics of liver injury and proposing recommendations for follow-up

**DOI:** 10.1186/s13023-020-01630-2

**Published:** 2021-01-07

**Authors:** Rodrigo Tzovenos Starosta, Suzanne Boyer, Shawn Tahata, Kimiyo Raymond, Hee Eun Lee, Lynne A. Wolfe, Christina Lam, Andrew C. Edmondson, Ida Vanessa Doederlein Schwartz, Eva Morava

**Affiliations:** 1 Graduate Program in Genetics and Molecular Biology, Universidade Federal do Rio Grande do Sul (UFRGS), Porto Alegre, RS Brazil; 2 Department of Clinical Genomics, Mayo Clinic, Rochester, MN USA; 3 Department of Pediatrics, Washington University in Saint Louis, St. Louis, MO USA; 4 Department of Internal Medicine, Mayo Clinic, Rochester, MN USA; 5 Biochemical Genetics Laboratory, Department of Laboratory Medicine and Pathology, Mayo Clinic, Rochester, MN USA; 6 Undiagnosed Diseases Program, Common Fund, National Institutes of Health, Bethesda, MD USA; 7 Division of Genetic Medicine, University of Washington, Seattle, WA USA; 8 Center of Integrated Brain Research, Seattle Children’s Research Institute, Seattle, WA USA; 9 Section of Biochemical Genetics, Division of Human Genetics, Department of Pediatrics, Children’s Hospital of Philadelphia, Philadelphia, PA USA; 10 Service of Medical Genetics, Hospital de Clínicas de Porto Alegre, UFRGS, Porto Alegre, RS Brazil; 11 Center for Individualized Medicine, Mayo Clinic, Rochester, MN USA

**Keywords:** CDG, Liver injury, Phosphomannomutase-2, Liver fibrosis, Cirrhosis, Phenotyping, Glycosylation

## Abstract

**Background:**

The congenital disorders of glycosylation (CDG) are a heterogeneous group of rare metabolic diseases with multi-system involvement. The liver phenotype of CDG varies not only according to the specific disorder, but also from patient to patient. In this study, we sought to identify common patterns of liver injury among patients with a broad spectrum of CDG, and to provide recommendations for follow-up in clinical practice.

**Methods:**

Patients were enrolled in the Frontiers in Congenital Disorders of Glycosylation natural history study. We analyzed clinical history, molecular genetics, serum markers of liver injury, liver ultrasonography and transient elastography, liver histopathology (when available), and clinical scores of 39 patients with 16 different CDG types (PMM2-CDG, n = 19), with a median age of 7 years (range: 10 months to 65 years). For patients with disorders which are treatable by specific interventions, we have added a description of liver parameters on treatment.

**Results:**

Our principal findings are (1) there is a clear pattern in the evolution of the hepatocellular injury markers alanine aminotransferase and aspartate aminotransferase according to age, especially in PMM2-CDG patients but also in other CDG-I, and that the cholangiocellular injury marker gamma-glutamyltransferase is not elevated in most patients, pointing to an exclusive hepatocellular origin of injury; (2) there is a dissociation between liver ultrasound and transient elastography regarding signs of liver fibrosis; (3) histopathological findings in liver tissue of PMM2-CDG patients include cytoplasmic glycogen deposits; and (4) most CDG types show more than one type of liver injury.

**Conclusions:**

Based on these findings, we recommend that all CDG patients have regular systematic, comprehensive screening for liver disease, including physical examination (for hepatomegaly and signs of liver failure), laboratory tests (serum alanine aminotransferase and aspartate aminotransferase), liver ultrasound (for steatosis and liver tumors), and liver elastography (for fibrosis).

## Introduction

The congenital disorders of glycosylation (CDG) are a group of rare inherited metabolic diseases, mostly autosomal recessive, that affect the complex process of building, remodeling, and transferring glycans to proteins and lipids. There are approximately 130 known CDG to date [1]. The current classification divides protein glycosylation according to which of the two main types of glycan attachment to proteins is affected [2]: N-linked or O-linked glycosylation. N-linked CDG are further subdivided based on whether the enzymatic defect impacts the assembly and transfer of primary glycan chains in the endoplasmic reticulum (CDG-I) or the maturation of glycan chains in the Golgi apparatus (CDG-II). Because of the ubiquity of glycosylation in human physiology [3], CDG tend to be multisystem diseases, affecting different organs and tissues in a heterogeneous way [4]. One of the most commonly affected organs in many CDG is the liver, due to its central role in protein secretion [4]; however, there is limited study of the liver phenotype of CDG beyond PMM2-CDG [5].

PMM2-CDG (MIM: #212065) is the most common CDG, with more than a thousand patients reported so far [6]. Most PMM2-CDG patients have mild liver dysfunction with increased serum aminotransferases, especially during the first 5 years of life, when all-cause lethality is also higher [5]; however, a proportion of patients develops steatosis, fibrosis/cirrhosis, and may die from the complications of liver failure [5]. Other CDG have more specific hepatic phenotypic characteristics. For instance, CCDC115-CDG (MIM: #616828) and MPI-CDG (MIM: #602579) have especially severe liver disease [7]. CCDC115-CDG presents with a Wilson disease-like phenotype with hepatosplenomegaly, increased serum aminotransferases, neonatal jaundice, early cirrhosis, and increased liver copper concentration [8–10]; other CDG involved with Golgi membrane trafficking such as TMEM199-CDG can also present with similar Wilsonian features [11, 12]. MPI-CDG tends to present with severe liver dysfunction and rapidly progressive (sometimes congenital) fibrosis [7, 13], although asymptomatic adults have been reported [14, 15]. Other CDG, such as ALG8-CDG (MIM: #603147), can present with severe liver disease with cirrhosis and complications of portal hypertension, with high lethality [7].

The hepatic phenotype in CDG is complex and understanding the manifestations and the type of liver injury to be expected in a patient is critical for appropriate management. In this study, we prospectively evaluate a large cohort of patients with different CDG types to systematically explore the hepatic phenotype of these disorders, seeking to identify common patterns of liver injury and to derive recommendations for clinical practice.

## Methods

### Subjects

Patients with a molecularly confirmed diagnosis of CDG and biochemical confirmation of the specific defect were recruited to the Frontiers of Congenital Disorders of Glycosylation (FCDGC) natural history study or to the CDG Nutritional Intervention study at Mayo Clinic. Retrospective and prospective data collection was part of the enrollment process, as the patients have been previously followed-up by standard of care.

### Data extraction

Blood tests were considered to be elevated when the results were above the reference levels on two or more occasions; in subjects having only one test, it was considered elevated if above the reference level in this measurement. The number of subjects with a given manifestation is represented over the number of subjects with an available result for such manifestation, not over the total number of subjects.

The following variables for the composite categorical parameters on extra-hepatic phenotype were used, being considered as positive when at least one of the variables in each parameter was abnormal: hematological abnormalities (hemoglobin, white blood cell count, platelet count); coagulation abnormalities (prothrombin time, activated partial thromboplastin time, factor XI activity, antithrombin III activity, clinically significant bleeding or thrombosis); dyslipidemia (low-density lipoprotein, high-density lipoprotein, total cholesterol, triglycerides); hypothyroidism (thyroid-stimulating hormone); glucose homeostasis (blood glucose, documented hypoglycemia); seizures (history of seizures, diagnosis of epilepsy). These categories were chosen based on the predominant extra-hepatic features of most CDG and on the availability of data.

The Nijmegen Pediatric CDG Rating Score (NPCRS) was performed in every patient in the study by the same investigator (EM). The NPCRS is a clinical tool developed as a means of quantifying clinical severity of patients with CDG in a global and comprehensive way, and is validated for all age groups [16].

### Statistical analysis

Categorical variables were compared using Fisher’s exact test with a significance level of *p* < 0.05. Q–Q plots were used to determine approximate parametricity. Parametric continuous variables were compared in an exploratory analysis between groups using Student’s *t*-test with a significance level of *p* < 0.05. A logistic regression was used to identify correlations of continuous variables with categorical outcomes, with a significance level of *p* < 0.05.

## Results

### Subjects

Thirty-nine patients were included in the study. Median age was 7 years (range: 10 months to 65 years), and 66.7% (26/39) were male (Table 1). The most common diagnosis was PMM2-CDG (n = 19); followed by ALG12-CDG (MIM: #607143), ALG13-CDG (MIM: #300884), DHDDS-CDG (MIM: #613861), PGM1-CDG (MIM: #614921), and SLC35A2-CDG (MIM: #300896) (n = 2 each); and ALG6-CDG, ALG8-CDG (MIM: #608104), CCDC115-CDG, DDOST-CDG (MIM: #614507), MPI-CDG, SLC10A7-CDG (MIM: #618363), SLC35C1-CDG (MIM: #266265), SLC39A8-CDG (MIM: #616721), TMEM165-CDG (MIM: #614727), and VMA21-CDG (MIM: #310440) (n = 1 each).

**Table 1:** Patient characteristics and markers of liver injury

Patient	Age (Y)	Sex	CDG	Genotype	Protein change	ALT range	AST range	AlkP range
1	1	M	PMM2-CDG	c.44G > C; c.422G > A	p.Gly15Ala; p.Arg141His	15–145	10–147	172–301
2	1	M	PMM2-CDG	c.357C > A; c.422G > A	p.Phe119Leu; p.Arg141His	103–571	93–829	172–304
3	2	M	PMM2-CDG	c.357C > A; c.422G > A	p.Phe119Leu; p.Arg141His	53–1595	54–1222	222–464
4	3	M	PMM2-CDG	c.422G > A; c.691G > A	p.Arg141His; p.Val231Met	48–66	54–57	173–182
5	5	M	PMM2-CDG	c.422G > A; c.548 T > C	p.Arg141His; p.Phe183Ser	34–41	23	186
6	5	F	PMM2-CDG	c.338C > T; c.710C > G	p.Phe113Leu; p.Thr234Arg	8–745	13–678	17–283
7	6	M	PMM2-CDG	c.357C > A; c.422G > A	p.Phe119Leu; p.Arg141His	25–1366	25–1986	115–1083
8	6	F	PMM2-CDG	c.415G > A; c.422G > A	p.Glu139Lys; p.Arg141His	19–31	31–48	157–250
9	6	M	PMM2-CDG	c.338C > T; c.422G > A	p.Pro113Leu; p.Asp148Asn	32–2524	26–4789	60–292
10	6	M	PMM2-CDG	c.422G > A; c.647A > T	p.Arg141His; p.Asn216Ile	37–71	45–69	104–155
11	7	F	PMM2-CDG	c.563A > G; c.691G > A	p.Asp188Gly; p.Val231Met	38–556	31–457	228–299
12	7	M	PMM2-CDG	c.205C > T;c.422G > A	p.Pro69Ser; p.Asp148Asn	28	42	225–242
13	8	M	PMM2-CDG	c.98A > C; c.140C > T	p.Gln33Pro; p.Ser47Leu	15–20	29–34	139–149
14	11	M	PMM2-CDG	c.422G > A; c.722G > C	p.Arg141His; p.Cys241Ser	17–18	25–26	154–172
15	12	M	PMM2-CDG	c.470 T > C; c.710C > T	p.Phe157Ser; p.Thr237Met	44–68	46–58	187–240
16	15	M	PMM2-CDG	c.422G > A; c.458 T > C	p.Arg141His; p.Ile153Thr	30–117	30–244	94–215
17	23	M	PMM2-CDG	c.26G > A; c.442G > A	p.Cys9Tyr; p.Asp148Asn	19–25	21–27	61–75
18	27	M	PMM2-CDG	c.357C > A; c.422G > A	p.Phe119Leu; p.Arg141His			
19	33	F	PMM2-CDG	c.357C > A; c.357C > A	p.Phe119Leu; p.Phe119Leu	14–164	21–327	44–48
20	31	M	ALG12-CDG	c.671C > T; c.1001delA	p.Thr224Met; p.Asn334ThrfsX15	18	23	61
21	46	M	ALG12-CDG	c.671C > T; c.1001delA	p.Thr224Met; p.Asn334ThrfsX15	15	22	121
22	1	F	ALG13-CDG	c.320A > G	p.Asn107Ser	10–18	40–49	102–165
23	4	F	ALG13-CDG	c.320A > G	p.Asn107Ser	17–49	28–51	118–405
24	59	M	DHDDS-CDG	c.124A > G; c.124A > G	p.Lys42Glu; p.Lys42Glu	25–27	37–38	77–82
25	63	F	DHDDS-CDG	c.124A > G; c.124A > G	p.Lys42Glu; p.Lys42Glu	20–27	28–33	69–82
26	2	M	PGM1-CDG	c.265G > A; c.988G > C	p.Gly89Arg; p.Gly330Arg	25	122	168
27	27	F	PGM1-CDG	c.206 T > C; c.313A > T	p.Met67Arg; p.Lys105X	26–61	46–236	53–61
28	1	F	SLC35A2-CDG	c.340A > T	p.Lys114X	8–35	33–78	83–235
29	12	F	SLC35A2-CDG	c.815G > A	p.Trp272X	12–87	17–61	147–177
30	21	F	ALG6-CDG	c.998C > T	p.Ala333Val	12–50	17–51	81–275
31	8	M	ALG8-CDG	c.584 T > C; c.1334 T > C	p.Leu195Pro; p.Leu445Pro	20–165	14–148	73–275
32	65	F	DDOST-CDG	c.20C > G; c.1325 T > A	p.Ala7Gly; p.Phe442Tyr	16–33	13–21	66–123
33	6	M	MPI-CDG	c.488-1G > C; c.656G > A	IVS4-1G > C; p.Arg219Gln	141–237	69–90	141–210
34	3	M	CCDC115-CDG	c.92 T > C; c.92 T > C	p.Leu31Ser; p.Leu31Ser	117–204	129–319	1071–1459
35	11	M	SLC10A7-CDG		Whole gene deletion (biallelic)	23	50–56	161–203
36	12	F	SLC35C1-CDG	c.503_505delTCT; c.942C > G	p.Phe168del; p.Tyr314X	17–29	19–29	211–273
37	2	M	SLC39A8-CDG	c.802C > T; c.802C > T	p.His268Tyr; p.His268Tyr	19–23	40–45	200–233
38	14	M	TMEM165-CDG	c.151C > T; c.725C > A	p.Gln51X; p.Thr242Lys	51–60	252–309	158–219
39	42	M	VMA21-CDG	c.52A > G	p.Arg18Gly*	24–48	39–62	113–160

Patients 20–21; 24–25 are siblings

*Y* years-old, *ALT* alanine aminotransferase, *AST* aspartate aminotransferase, *AlkP* alkaline phosphatase, *M* male, *F* female. Age displayed is the current age. Normal ranges: ALT < 42 IU/L; AST < 41 IU/L; AlkP < 300 IU/L

### Clinical findings

Clinical descriptions were available for all patients. Six patients had hepatomegaly on physical exam (patients 2, 6, 8, 11, 23, and 34). Three patients had neonatal jaundice requiring phototherapy (patients 10, 12 and 26), but no patient had jaundice outside of the neonatal period. One patient had ascites (patient 6); no patient had other stigmata of liver disease (e.g., *caput medusae*, gynecomastia, or palmar erythema) on physical exam, although one patient (patient 7) had unexplained pruritus. Patient 6 underwent liver transplantation at 4 years of age because of liver failure (hyperammonemia, recurrent ascites).

### Molecular findings

All patients had genetically confirmed CDG genotypes. The most common genetic variant found in *PMM2* was c.422G > A (p.Arg141His), comprising 14/38 (36.8%) of alleles reported (Table 1). The only homozygous *PMM2* variant found was c.357C > A (p.Phe119Leu). Two different *PMM2* variants were found to arise in the same nucleotide position: c.710C > G (p.Thr234Arg) and c.710C > T (p.Thr234Met).

### Laboratory findings: liver markers

**Fig. 1:**
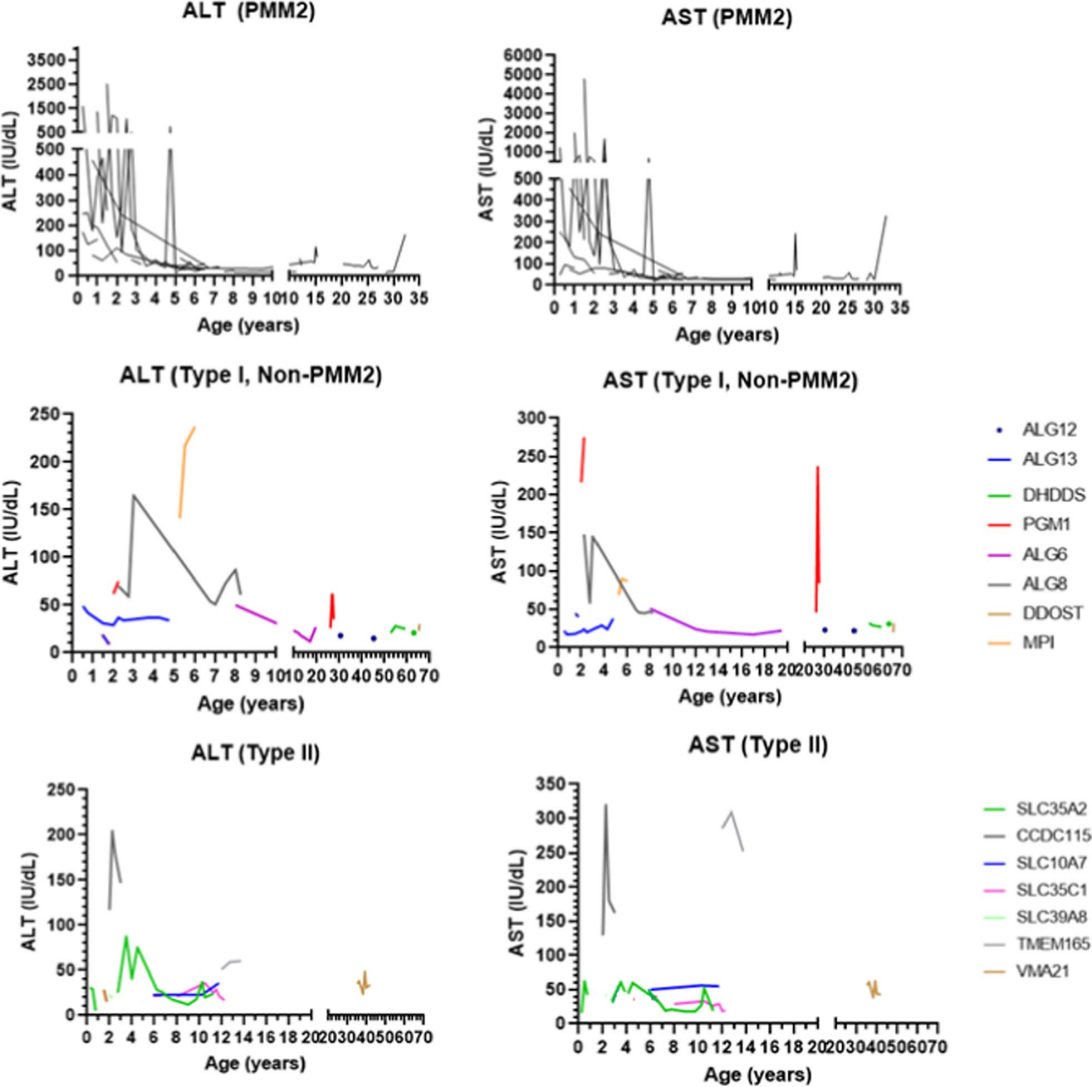
Aminotransferase values in all CDG patients according to type (**a** ALT values in PMM2-CDG patients. **b** AST values in PMM2-CDG patients. **c** ALT values in non-PMM2-CDG CDG-I patients. **d** AST values in non-PMM2-CDG CDG-I patients. **e** ALT values in CDG-II patients. **f** AST values in CDG-II patients). There is a notable inflexion point around 5 years of age in the figures A and B, after which most values tend to be normal or near-normal. A less defined inflexion point can be noted in the figures C and D at approximately 8 years of age, although there are still many patients with elevated values after this age

Liver markers for all patients are shown in detail in Table 1. Alanine-aminotransferase (ALT) values were available for 37 patients: of these, 18 (48.6%) had elevated values (normal < 42 IU/L). Aspartate-aminotransferase (AST) values were available for 37 patients; of these, 26 (70.3%) had elevated values (normal < 41 IU/L). Elevations in both enzymes were found in 18/37 patients (48.6%). Gamma-glutamyltransferase (GGT) values were available for 16 patients; of these, 4 (25%) had elevated values (patient 6: 157 IU/L; patient 7: 45 IU/L; patient 9: 57 IU/L; patient 27: 56 IU/L; normal < 41 IU/L). Alkaline phosphatase (AlkP) values were available for 37 patients; of these, 3 (8.1%) had elevated values (normal < 300 IU/L). Total bilirubin values were available for 36 patients; of these, only 1 had elevated values (patient 32: maximum value 1.5 mg/dL, no direct/indirect differential; normal total bilirubin < 1.3 mg/dL). Aminotransferase values according to age are displayed in Fig. 1 A-F.

In PMM2-CDG patients, no association was observed between the frequency of altered aminotransferase values and the presence of the most common *PMM2* variant, c.422G > A (p.Arg141His) (ALT, p = 0.604; AST, p = 0.518).

### Liver ultrasound and transient elastography (FibroScan) findings

Liver ultrasound results were available for 23 patients (Table 2). The most common finding was coarse hepatic echotexture (6/24, 26.1%). Four patients (17.4%) had steatosis and two patients (8.7%) had focal lesions (one diagnosed with a solitary hemangioma of the liver; the other with multiple circumscribed echogenic lesions with no blood flow on color imaging, the largest being subcapsular and measuring 6 × 7 × 9 mm). Seven patients (30.4%) had normal liver ultrasounds.

**Table 2:** Patients that underwent liver ultrasound or transient elastography

Patient	CDG	Age at ultrasound (Y)	Ultrasound findings	Transient elastography result
1	PMM2	1	Coarse liver parenchyma	F0 (3.8 kPa)
2	PMM2	1	Coarse liver parenchyma	–
3	PMM2	1	Steatosis^a^	–
6	PMM2	4	Coarse liver parenchyma	–
7	PMM2	5	Coarse liver parenchyma	–
8	PMM2	6	Central intrahepatic bile duct dilation, otherwise normal	F2 (8.4 kPa)
9	PMM2	6	Normal	–
11	PMM2	1	Normal	–
12	PMM2	5	Hemangioma, otherwise normal	F1 (7.3 kPa)
14	PMM2	6	“Starry sky” parenchyma	–
15	PMM2	10	Normal	–
17	PMM2	15	Steatosis	
19	PMM2	28	Coarse liver parenchyma	–
22	ALG13	1	Geographic steatosis	–
23	ALG13	4	Multiple well-circumscribed echogenic lesions	–
25	DHDDS	63	Normal	–
27	PGM1	2	Increased echogenicity	
28	SLC35A2	4 days	Normal	–
31	ALG8	6	Steatosis	–
33	MPI	–	–	F2 (8.0 kPa)
34	CCDC115	2	Mildly increased echogenicity	–
37	SLC39A8	2	Coarse liver parenchyma	–
38	TMEM165	12	Normal	–
39	VMA21	34	Normal	–

^a^Resolved subsequently

Four patients had transient hepatic elastography (Table 2); of these, 3 had also liver ultrasounds. One patient who had coarse liver parenchyma on ultrasound had a METAVIR score [17] of F0 on elastography, indicating no fibrosis. On the other hand, two patients with no signs of fibrosis on ultrasound had METAVIR scores of F1 and F2 on elastography, indicating incipient fibrosis.

### Liver biopsy

**Fig. 2:**
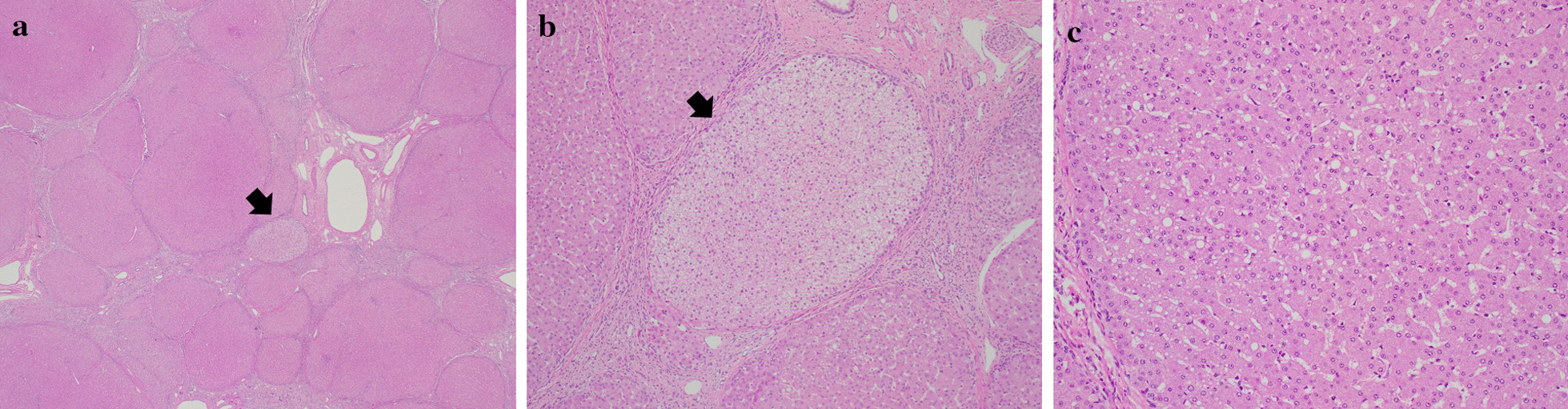
**a** liver, hematoxylin and eosin, 20 ×. Cirrhotic liver tissue with extensive bridging and enlarged portal tract. There is focal glycogen deposition in the cytoplasm of hepatocytes of a nodule (black arrow). **b** Liver, hematoxylin and eosin, 200 ×. Hepatocellular nodule with cytoplasmic glycogen deposition (black arrow). **c** Liver, hematoxylin and eosin, 200 ×. Hepatocellular nodule with discrete macrovesicular steatosis

Histopathological reports for liver tissue were available for three patients: two patients underwent liver biopsy and one had an explanted liver. The liver explanted from patient 6 (PMM2-CDG) showed micronodular cirrhosis on gross and microscopic examination (Fig. 2); there was mild macrovesicular steatosis and mild-to-moderate biliary ductular proliferation, as well as multiple foci of hepatocellular glycogen storage in the cytoplasm; iron staining was negative. Patient 7 (PMM2-CDG) had a liver biopsy at 3 years of age that showed broad, irregular bridging fibrosis with minimal mixed (macrovesicular and microvesicular) steatosis and increased hepatocellular glycogen, with no signs of biliary changes, iron overload, or hepatocellular regeneration. Patient 34 (CCDC115-CDG) had a liver biopsy at 2 years of age that showed cirrhosis with extensive portal and periportal bridging fibrosis, microvesicular steatosis, positive glycogen staining with fine hepatocellular cytoplasm vacuolization in a mosaic pattern, with no signs of biliary changes or iron overload. All glycogen deposits were confirmed by positivity on periodic acid Schiff (PAS) staining, with attenuation after diastase treatment.

### Treatment effects

**Fig. 3:**
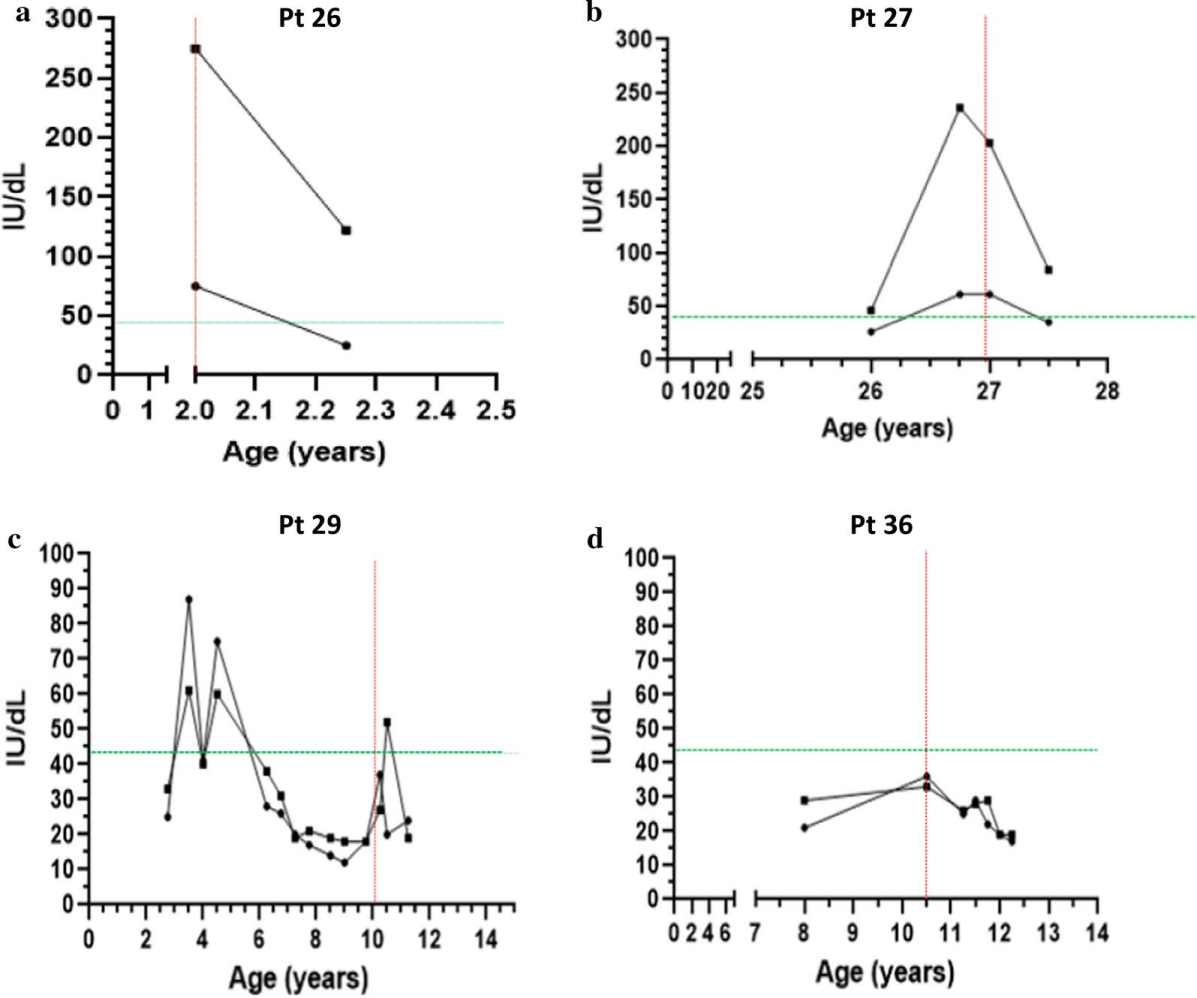
Evolution of ALT (circles) and AST (squares) values in treated patients. Red line = onset of treatment; green line = upper limit of normal. **a**, **b** Improvement after initiation of oral galactose therapy in two patients with PGM1-CDG; **c** mild transient elevation of aminotransferase values after initiation of oral galactose therapy in a patient with SLC35A2-CDG, with rapid normalization; **d** absence of significant change from a normal baseline after initiation of oral fucose therapy in a patient with SLC35C1-CDG

Eight patients underwent specific monosaccharide supplementation therapy for CDG in this study. Patient 26 with PGM1-CDG started treatment with oral galactose 0.5 g/kg/day at age 2 years, with a planned increase to 1 g/kg/day after 6 weeks of the escalating dose. ALT and AST were elevated before treatment, with a rapid decrease (3 weeks) after introduction of treatment. (Fig. 3a). Patient 27 with PGM1-CDG started treatment with oral galactose 0.5 g/kg/day at age 27 years, with a significant decrease in AST and ALT within 6 months after introduction of treatment (Fig. 3b). Patient 28 with SLC35A2-CDG started treatment with oral galactose 1.5 g/kg/day at age 6 months. AST and GGT were elevated before treatment, which both normalized on treatment within 4 months, and additionally, significant improvement was observed in other clinical features such as seizure control and motor skills. Patient 29 with SLC35A2-CDG started treatment with oral galactose 1.5 g/kg/day at age 10 years. There was a mild transient elevation in ALT and AST levels after initiation of treatment (Fig. 3c). Although transaminases normalized after the initial increase, no significant improvement was observed in clinical features during the first 4 months of therapy, as described elsewhere [18]. Patient 33 with MPI-CDG has been on treatment with oral mannose 1 g/kg/day divided in 6 doses at age 4 years for 2 years. High aminotransferase values were observed during the whole period of 2 years of treatment and the patient needed additional nutritional intervention with a complex carbohydrate diet. Patient 36 with SLC35C1-CDG started treatment with oral fucose 2 g/day at age 10 years, with an increase to 4 g after 6 months of therapy and 8 g/day at age 11 years. There was no elevation of ALT, AST, or AlkP before or during treatment (Fig. 3d), although clinical parameters such as tendency to infections were improved. Patient 37 with SLC39A8-CDG started treatments with oral galactose 1 g/kg/day at age 2 years and escalating doses of manganese from 5 mg/day to 65 mg/day during a period of 6 months. No significant change was observed in ALT and AST levels after introduction of treatment; however, the patient had significant clinical improvement with respect to seizure control and motor development over the first 6 months of therapy. Patient 38 with TMEM165-CDG started treatment with oral galactose 1.5 g/kg/day at age 5 years. No pre-treatment laboratory results are available. High aminotransferase values were observed during treatment.

### Nijmegen Pediatric CDG rating scale (NPCRS)

A NPCRS [16] score was available for 38 patients. The total median was 21, ranging from 4 to 40. NPCRS medians (range) by CDG subgroup were: PMM2-CDG = 24 (15–40); non-PMM2 type I CDG = 17.5 (4–27); for type II CDG = 25 (4–35). A Q-Q plot determined the distribution of NPCRS values to be approximately normal. There was no difference in the total NPCRS values between patients with elevated ALT and patients with normal ALT (*p* = 0.215). A significant difference was found in the NPCRS scores between patients with elevated and normal AST values, with patients with elevated AST measurements having higher NPCRS values (respectively, 23.46 ± 8.04 vs 15.60 ± 6.83, *p* = 0.009). A binary logistic regression between having high AST values and the tree sections of the NPCRS showed that this significant association is due to the first section (“current function”) only, with a *p* = 0.045, r^2^ = 0.353. When analyzing only CDG-I patients, we identified that increases in both ALT and AST are associated with higher NPCRS scores (ALT: 23.52 ± 7.57 vs 17.25 ± 6.52, *p *= 0.028; AST: 23.15 ± 7.03 vs 16.00 ± 7.12, *p* = 0.018); a binary logistic regression showed that this significant association is due to the second section for ALT (*p* = 0.022, r^2^ = 0.412), but to no specific section for AST (lowest *p* = 0.094). There was no association with a higher rate of elevated ALT or AST and a diagnosis of PMM2-CDG versus another type I CDG (ALT, *p* = 0.148; AST, *p* = 0.422). On a subgroup analysis (grouping patients according to specific CDG), however, we did not find any significant difference in total NPCRS scores or sections.

Patients with PMM2-CDG and a diagnosis of coarse liver parenchyma on ultrasound had a non-significantly higher NPCRS than PMM2-CDG patients who did not have this diagnosis (29.00 ± 8.28 vs 23.37 ± 5.15, *p* = 0.173).

### Extra-hepatic associations

Prevalence of extra-hepatic manifestations of CDG is displayed in Table 3. A significant association was found in the co-distribution of high ALT levels and abnormal coagulation parameters (85% of patients with high ALT levels had coagulation abnormalities vs 22.7% of patients with coagulation abnormalities with normal ALT, *p* = 0.002) and hypothyroidism, as defined by elevated TSH measurements (all patients with hypothyroidism had high ALT values vs 41.3% of patients without hypothyroidism having high ALT values, *p* = 0.004). No association was found in the co-distribution of high ALT levels and abnormal hematological parameters, dyslipidemia, glucose homeostasis, or seizures. For high AST values, a significant association was found for coagulation parameters (73.1% of patients with high AST levels had coagulation abnormalities vs 13.6% of patients who had normal AST values, *p* = 0.026) and seizures (65.5% of patients with high AST levels had seizures vs 13.6% of patients who had normal AST values, *p* = 0.037).

**Table 3:** Prevalence of extra-hepatic manifestations in CDG patients

CDG	Hematological abnormalities	Coagulation abnormalities	Dyslipidemia	Hypothyroidism	Glucose homeostasis	Seizures
PMM2	3/15	12/15	4/11	6/15	8/13	8/16
ALG6	N/A	1/1	0/1	0/1	0/1	1/1
ALG8	0/1	1/1	N/A	0/1	0/1	1/1
ALG12	N/A	2/2	0/2	0/2	0/2	0/2
ALG13	0/2	0/2	1/1	0/2	0/2	2/2
CCDC115	0/1	0/1	1/1	N/A	0/1	0/1
DDOST	0/1	0/1	1/1	0/1	1/1	0/1
DHDDS	1/2	0/2	2/2	0/2	2/2	1/2
MPI	0/1	1/1	N/A	1/1	1/1	1/1
PGM1	1/2	2/2	0/1	1/2	2/2	0/2
SLC10A7	0/1	1/1	1/1	0/1	0/1	0/1
SLC35A2	1/2	0/2	1/1	0/2	1/2	2/2
SLC35C1	0/1	N/A	N/A	0/1	0/1	0/1
SLC39A8	0/1	0/1	N/A	0/1	0/1	1/1
TMEM165	0/1	0/1	1/1	0/1	0/1	1/1
VMA21	0/1	0/1	1/1	0/1	0/1	0/1

Values are displayed as the number of patients with the manifestation divided by the number of patients for whom there was available data regarding the manifestation

## Discussion

The liver is one of the main sites of N-glycosylation in the body [19] and is responsible for the attachment of glycans to most secreted proteins. CDG affect the liver in a number of ways. In this retrospective/prospective study we have described a cohort of patients with 16 different CDG and confirmed [7] that serum aminotransferases, which are common, reliable markers of hepatocellular injury, are mostly elevated during the first 5 years of life in most types of CDG but improve significantly after this age. Exceptions to this in our study were ALG8-CDG, CCDC115-CDG, MPI-CDG, PGM1-CDG, and TMEM165-CDG patients. In contrast to aminotransferases, we found that few patients had an elevation of the cholangiocyte injury marker GGT, and that most of such elevations were mild. Normal GGT in the setting of elevated aminotransferases had previously been reported in isolated cases of CDG [8, 13], but to the best of our knowledge this is the first time it has been reported systematically. This highlights the hepatocellular, rather than global, nature of liver damage in this group of disorders.

The most common finding on liver ultrasound was a coarse liver parenchyma, which is usually associated with fibrotic changes. However, we have observed a disparity between liver ultrasound and transient elastography, a fibrosis-directed measurement. Unfortunately, we only have data from a few patients on liver elastography, because elastography has not been considered as standard-of-care in pediatric patients with CDG, and these studies were performed prior to the study recruitment. Nonetheless, the disparity between elastography and liver ultrasound results indicates that both liver ultrasound and elastography should be used for follow-up in patients in CDG.

When analyzing the relationship between elevations of serum aminotransferases and extra-hepatic manifestations, we found that both elevated ALT and AST are associated with the presence of coagulation factor abnormalities. Since most coagulation factors are glycosylated in the liver, these changes mirror the degree of glycosylation abnormality. This highlights the necessity of screening for coagulopathy in patients with high aminotransferases in this population; however, it must be remembered that it is possible to find abnormal activity levels of coagulation factors and inhibitors with normal aminotransferase levels.

We had access to specimens of liver tissue from 3 patients. In the two PMM2-CDG patients, there were increased cytoplasmic glycogen deposits. In one of these patients this findings was in the context of liver cirrhosis and may be considered as a part of the cirrhotic phenotype [20]. The presence of scattered hepatocytes with foci of glycogenosis in the other may suggest a disturbance of hepatic glycogen breakdown which could stem from excessive mannose-6-phosphate being shunted to fructose-6-phosphate by mannose-phosphate isomerase [21] and feeding into glycolysis, which can inhibit glycogenolysis by ultimately increasing intracellular adenosine triphosphate. In the biopsy of the patient with CCDC115-CDG we could not observe any signs of cholestasis, foamy histiocytes, or necrotic lesions, as described [8, 9]. This highlights the heterogeneity of manifestations within a single type of CDG and depending on age.

We have observed different effects of short-term monosaccharide treatment according to the type of CDG. For PGM1-CDG, there was a rapid improvement in aminotransferase levels after introduction of galactose therapy, as expected [22–24]. For SLC35A2-CDG aminotransferases were mildly elevated before treatment in one patient, with improvement after galactose was initiated; and a mild transient elevation in aminotransferases in the other with normalization shortly thereafter. We did also notice improvement in neurological features (data not shown) as expected [18, 24, 25]. In MPI-CDG and TMEM165-CDG, mannose and galactose supplementation, respectively, do not always fully correct or prevent liver damage [13, 26, 27], and our observations match this expectation. SLC35C1-CDG affects primarily leukocytes and the central nervous system [28], and therapeutic goals with fucose supplementation involve restoration of immune function and improvement of neurological function [29]. SLC39A8-CDG affects primarily bone development and central nervous system function, without significant hepatic findings [30], in line with our findings.

Recently, Ferreira et al. proposed a categorization of metabolic liver disease according to the type of liver manifestation [31] including diseases with hepatomegaly, hepatocellular disease with elevation of aminotransferases or liver failure, cholestasis, steatosis, fibrosis, and liver tumors. Most CDG span more than one category (Tables 4, 5). This warrants the recommendation that every CDG patient be systematically evaluated for different types of liver disease with a combination of physical exam, laboratory testing, ultrasonography, and elastography.

**Table 4:** Distribution of liver manifestations per CDG in this study

CDG	Hepatomegaly	Hepatocellular disease	Cholestasis	Steatosis	Fibrosis	Liver tumor
PMM2	+	+	+	+	+	+
ALG6	−	+	−	N/A	N/A	−
ALG8	−	+	−	+	−	−
ALG12	−	−	−	N/A	N/A	−
ALG13	+	+	−	+	−	+
CCDC115	+	+	−	+	+	−
DDOST	−	−	+	N/A	N/A	−
DHDDS	−	−	−	−	−	−
MPI	−	+	−	N/A	+	−
PGM1	−	+	−	N/A	N/A	−
SLC10A7	−	+	−	N/A	N/A	−
SLC35A2	−	+	−	−	−	−
SLC35C1	−	−	−	N/A	N/A	−
SLC39A8	−	−	−	−	−	−
TMEM165	−	+	−	−	−	−
VMA21	−	+	−	−	−	−

Classification adapted from Ferreira et al. [31]. Hepatomegaly was defined as an enlarged liver on physical exam or ultrasonography. Hepatocellular disease was defined as elevated ALT or AST. Cholestasis was defined as jaundice, elevated bilirubin, or histopathological evidence of bile accumulation. Steatosis was defined as ultrasonographic or histopathological evidence of hepatocellular lipid accumulation. Fibrosis was defined as transient elastography score equal or greater than F1 or histopathological evidence of fibrosis. Liver tumor was defined as any imaging evidence of tumoral growth (neoplastic or non-neoplastic) in the liver. + denotes presence of the manifestation in the phenotype of at least one patient with the disorder; − denotes absence of the manifestation in all patients analyzed

**Table 5:** Distribution of liver manifestations by CDG as reported in the literature

CDG	Hepatomegaly	Hepatocellular disease	Cholestasis	Steatosis	Fibrosis	Liver tumor	References
PMM2	+	+	+	+	+	−	[5–7]
ALG6	+	+	+	−	−	−	[7, 32]
ALG8	+	+	+	+	+	−	[7]
ALG12	−	+	−	−	−	−	[33]
ALG13	+	−	−	−	−	−	[34]
CCDC115	+	+	+	+	+	−	[7]
DDOST	−	+	−	−	−	−	[35]
DHDDS	+	+	+	−	−	−	[36]
MPI	+	+	−	+	+	−	[37]
PGM1	+	+	−	+	+	−	[7]
SLC10A7	−	−	−	−	−	−	
SLC35A2	−	+	−	−	−	−	[38, 39]
SLC35C1	−	−	−	−	−	−	
SLC39A8	−	−	−	−	−	−	
TMEM165	+	+	−	−	−	−	[40]
VMA21	−	+	+	+	−	−	[41]

Classification adapted from Ferreira et al. [31]. Hepatomegaly was defined as an enlarged liver on physical exam or ultrasonography. Hepatocellular disease was defined as elevated ALT or AST. Cholestasis was defined as jaundice, elevated bilirubin, or histopathological evidence of bile accumulation. Steatosis was defined as ultrasonographic or histopathological evidence of hepatocellular lipid accumulation. Fibrosis was defined as transient elastography score equal or greater than F1 or histopathological evidence of fibrosis. Liver tumor was defined as any imaging evidence of tumoral growth (neoplastic or non-neoplastic) in the liver. + denotes presence of the manifestation in the phenotype of at least one patient with the disorder; − denotes absence of the manifestation in all patients analyzed. The column “References” refer to the positive findings in each disorder

## Conclusion

In summary, in our retrospective/prospective dataset of CDG patients we confirm spontaneous improvement of hepatocellular injury markers AST and ALT in CDG-I but not in CDG-II. There was a dissociation between liver ultrasound and transient elastography results in several patients necessitating prospective liver elastography in CDG patients to survey for possible liver fibrosis. The cytoplasmic glycogen deposits found in liver biopsy specimens raise the hypothesis that a disturbance of glycogen metabolism may be present in PMM2-CDG, leading to a need of further investigation. Based on our findings, we recommend that all CDG patients have regular systematic, comprehensive screening for liver disease, including clinical, laboratory and imaging techniques that detect both steatosis and fibrosis.

## Abbreviations

AlkP: Alkaline phosphatase
ALT: Alanine aminotransferase
AST: Aspartate aminotransferase
CDG: Congenital disorders of glycosylation
FCDGC: Frontiers in congenital disorders of glycosylation
GGT: Gamma glutamyltransferase
MIM: Mendelian inheritance on man
NPCRS: Nijmegen pediatric CDG rating score
PAS: Periodic acid Schiff
TSH: Thyroid stimulating hormone

## Availability of data and material

All data generated or analyzed during this study are included in this published article.
